# Intestinal nutrition: role of vitamins and biofactors and gaps of knowledge

**DOI:** 10.1016/j.psj.2021.101665

**Published:** 2021-12-30

**Authors:** Douglas R. Korver

**Affiliations:** Department of Agricultural, Food, and Nutritional Science, University of Alberta, Edmonton, Canada T6G 2P5

**Keywords:** poultry, vitamin, biofactor, intestinal health, nutrition

## Abstract

The role of the microbiota in the health of the host is complex and multifactorial. The microbiota both consumes nutrients in competition with the host, but also creates nutrients that can be used by other microbes, but also the host. However, the quantitative impact of the microbiota on nutrient supply and demand is not well understood in poultry. The gastrointestinal tract is one of the largest points of contact with the external environment, and the intestinal microbiome is the largest and most complex of any system. Although the intestinal microbiota has first access to consumed nutrients, including vitamins, and is potentially a major contributor to production of various vitamins, the quantification of these impacts remains very poorly understood in poultry. Based on the human literature, it is clear that vitamin deficiencies can have systemic effects on the regulation of many physiological systems, beyond the immediate, direct nutrient functions of the vitamins. The impact of excessive supplementation of vitamins on the microbiota is not well understood in any species. In the context of poultry nutrition, in which substantial dietary excesses of most vitamins are provided, this represents a knowledge gap. Given the paucity of studies investigating the vitamin requirements of modern, high-producing poultry, the limited understanding of vitamin nutrition (supply and utilization) by the microbiome, and the potential impacts on the microbiome of the move away from dietary growth-promoting antibiotic use, more research in this area is required.

The microbiota also contributes a vast array of other metabolites involved in intramicrobiota communication, symbiosis and competition that can also have an impact on the host. Myo-inositol and butyrate are briefly discussed as examples of biofactors produced by the microbiota as mediators of intestinal health.

## INTRODUCTION

Genetic selection of broiler chickens (Zuidhof et al., 2014), laying hens (Preisinger, 2018), and turkeys (Havenstein et al., 2007; Clark et al., 2019) has led to dramatically increased rates of production with a concomitant reduction in the amount of feed consumed per unit of production. Increases in productivity may place additional metabolic stress on the birds, and increase the requirement for several vitamins. The reduction or removal of antibiotic growth promotors from the diets of meat-type chickens and turkeys also has implications for the intestinal environment and vitamin nutrition (Feye et al., 2020), and likely the subsequent use and production of vitamins by the intestinal microbiota. Finally, the majority of vitamin requirement studies were published well before the most recent NRC Nutrient Requirements of Poultry (National Research Council, 1994) was released, used less productive bird genetics, and used predominantly semi-purified or purified diets (Leeson, 2007; National Research Council, 1994). Therefore, the relevance of such studies to modern poultry production may be limited. In general, a surfeit of each vitamin is provided in commercial poultry diets to overcome potential losses during feed manufacture and storage, and vitamin deficiencies for productivity are, therefore, not likely in the absence of formulation or mixing errors. However, with the expanding understanding of the importance of the gut microbiome on intestinal and whole-body health, a re-examination of vitamin nutrition in poultry is warranted. This review will provide a brief overview of vitamin use in commercial poultry production, introduce some examples of how new discoveries in nutrition may influence our understanding of vitamin metabolism in the gut, and identify knowledge gaps for which research is necessary. The production of 2 biofactors (myo-inositol and butyrate) by the gut microflora is discussed as further examples of microbially derived products that can influence the host. Note that inclusion of a particular company's recommendations or feeding guide in this review does not imply an endorsement, nor does exclusion reflect a bias against a competitor.

## CHANGES IN NUTRIENT REQUIREMENTS WITH GENETIC SELECTION FOR PRODUCTIVITY

### Genetic Selection for Increased Poultry Productivity

Genetic selection has resulted in a dramatic increase in growth rate in broilers chickens and turkeys, and egg production in laying hens. In each case, the increase in productivity has also been accompanied by an increase in the efficiency of feed utilization. Therefore, less feed is required per unit of body weight gain or egg production than in the past. Assuming a constant inclusion of each vitamin per kg of feed, it follows that vitamin intake per unit of productivity will decrease. Between 1985 and 2005, vitamin E intake per unit of productivity for layers, broilers and turkeys was estimated to have decreased by 1.1, 0.8, and 0.6% per year, assuming a constant feed vitamin E level of 20 IU/kg feed (Leeson, 2007). Since 2005, poultry productivity and feed efficiency have continued to increase. Although commercial feeding recommendations for vitamins have generally increased since that time (Table 1), the published vitamin requirements available at the time this paper was written (National Research Council, 1994) are likely woefully out of date. In spite of that, very little recent work has been conducted to determine the requirements for most vitamins for modern, highly productive and efficient poultry genetics.

**Table 1:** Vitamin requirements (units/kg feed) according to NRC (1994) compared to commercial recommendations. Modified from Leeson (2007).

	Laying hen^1^ (n)				Broiler starter				Turkey starter			
Vitamin, units/kg feed	NRC	Leeson & Summers^2^	Commercial^3^	Fold-excess^4^	NRC	Leeson & Summers^2^	Commercial^3^	Fold-excess^4^	NRC	Leeson & Summers^2^	Commercial^3^	Fold-excess^4^
Vitamin A (IU)	2,500	8,000	8,000–12,000	4.8	1,500	8,000	12,000–15,000	10.0	5,000	10,000	12,000–15,000	3.0
Vitamin D_3_ (IU)	250	3,500	3,000–4,000 plus 2,670 as 25-OH vitamin D_3_	26.7	200	3,500	4,000–5,000 plus 2,670 as 25-OH vitamin D_3_	38.4	1,100	3,500	4,000–5,000 plus 3,680 as 25-OH vitamin D_3_	7.0
Vitamin E (IU)	4	50	20–30	7.5	10	50	150–200	20.0	12	100	150–200	16.7
Vitamin K (IU)	0.4	3	2.5–3.0	7.5	0.5	3	3–4	8.0	1.75	3	4–5	2.9
Thiamine (mg)	0.6	2	2.5–3.0	5.0	1.8	4	3–4	2.2	2.0	3	4.5–5.0	2.5
Riboflavin (mg)	2.1	5	5–7	3.3	8.6	5	8–10	1.2	4.0	10	15–20	5.0
Niacin (mg)	8.3	40	30–50	6.0	35	40	60–80	2.3	60	60	100–150	2.5
Pantothenate (mg)	1.7	10	8–12	7.1	10	14	15–20	2.0	10.0	18	30–35	3.5
Pyridoxine (mg)	2.1	3	3.5–5	2.4	3.5	4	4–6	1.7	4.5	6	6–7	1.6
Biotin (µg)	80	100	100–150	1.9	150	100	250–400	2.7	250	250	250–400	1.6
Folacin (mg)	0.21	1	1.0–1.5	7.1	0.55	1	2–2.5	4.5	1.0	2	4–6	6.0
Vitamin B_12_ (µg)	4	10	15–25	6.3	10	12	20–40	4.0	3	20	40–50	16.7
Choline (mg)	875	400	300–500	0.6	1,300	400	400–700	0.5	1,600	800	1,000–1,200	0.8
Vitamin C^5^	–	–	100–200	–	–	–	100–200	–	–	–	100–200	–

1 Assuming 100 g/b/d feed consumption.

2 Leeson and Summers (2005).

3 DSM Nutritional Products (2016).

4 Commercial (DSM Nutritional Products, 2016) vs. NRC (1994). When a range is given in the table; the higher value was used to calculate the fold-excess.

5 No vitamin C requirement provided by NRC (1994) or Leeson and Summers (2005).

Genetic selection for increased productivity and feed efficiency may have also come with an increase in the ability of the bird to absorb nutrients. For example, the calcium requirement of 0.9% of the diet (National Research Council, 1994) for grower chickens was well in excess of the determined Ca requirement for BW gain, FCR, and tibia ash in a more recent broiler strain (Driver et al., 2005). This suggests that modern broiler chickens have a reduced dietary Ca requirement relative to older genetics, although it is not clear whether this is due to a truly reduced requirement, or an increase in absorptive efficiency. Even less work has been done investigating the efficiency of dietary vitamin absorption and utilization in modern poultry genotypes, and comparisons of vitamin requirements of historical and modern genotypes is similarly lacking.

### Safety Margins for Dietary Vitamin Inclusion

Nutrient requirements published in the 1994 NRC Nutrient Requirements of Poultry represent the minimum amount of dietary inclusion to reach a plateau in some outcome of interest (typically performance-related). The values are based on the total dietary content (intrinsic levels plus supplements to the diet). Historically, substantial safety margins relative to the NRC (1994) requirements have been included for vitamin supplementation of poultry diets. This is because the values published in that document represent the minimum amount of a vitamin to obtain a plateau in a specific outcome. These studies are often conducted under highly controlled, low-stress conditions, using purified or semi-purified diets that are not reflective of commercial conditions. A survey of 25 commercial Spanish vitamin premix manufacturers showed that safety margins (relative to NRC, 1994) for supplemental vitamins in broiler diets ranged from 2.1- to 13.7-fold for fat-soluble vitamins, and 0.5- to 2.1-fold for water-soluble vitamins excluding choline (Villamide and Fraga, 1999). The supplementation rates below the NRC (1994) requirement for some vitamins represent the vitamin contributions provided by the other dietary ingredients such as cereal grains and oilseed meals.

Leeson (2007) compared vitamin requirements and recommendations for broilers and layers from the National Research Council (1994), a poultry nutrition textbook (Leeson and Summers, 2005), and the contemporary recommendations of a commercial vitamin supplier. That approach has been updated in Table 1, and a calculation of the fold-excess in levels of dietary vitamins calculated as the high range for current commercial recommendations (DSM Nutritional Products, 2016) relative to the National Research Council (1994). Vitamin D recommendations have the greatest safety margin for layers and broilers (26.7- and 38.4-fold, respectively). Because of a higher minimum vitamin D requirement for turkeys, the excess is only 7-fold. Aside from vitamin D, the safety margins for laying hens range from 1.9-fold for biotin to 7.5-fold for vitamins E and K. Commercial vitamin E recommendations for broilers are 20-fold relative to NRC (1994), but recommendations for the other vitamins range from 1.2- to 8.0-fold greater than NRC (1994). Commercial turkey vitamin recommendations include 16.7-fold excesses relative to NRC (1994) for vitamin E and vitamin B_12_. Excesses for other vitamins range from 1.6- to 6.0-fold. Commercial recommendations for choline tend to be lower than the NRC recommendation (0.5- to 0.8-fold of the NRC, 1994 requirement), likely due to the relatively substantial contribution of choline from other dietary ingredients (National Research Council, 1994).

Because they are included at very low levels, vitamins contribute a relatively small amount to the overall cost of the diet. Compared to amino acid supplementation (Pack et al., 2003; Kidd and Tillman, 2016), therefore, there is much less economic incentive to more precisely match dietary supply with exact requirements.

The cost of individual vitamin analysis also contributes to the lack of precision in dietary vitamin supply. Vitamins are categorized together not because of structural or functional similarities, but because they do not fit within the other nutrient categories, but are required for life. Combs and McClung (2017) defined vitamins according to the following criteria:

• Organic compounds that are not fats, carbohydrates, or proteins
• Natural components of feeds, usually present in very small amounts
• Essential for normal physiological function, also usually in very small amounts
• Prevent specific deficiency signs, which occur when absent or poorly available
• Not synthesized by the host in sufficient quantities to meet physiological needs.

The vitamins do not necessarily share structural or physicochemical similarities, and therefore require multiple analyses for quantification in complete feeds or feedstuffs, typically by HPLC (Cortes-Herrera et al., 2018). Therefore, determining exact quantities present is expensive and time-consuming, limitations that can more efficiently be overcome by simply feeding the vitamins in excess of requirements.

Safety margins are also included in poultry diets because of the potential for loss of vitamin activity due to extended storage time, especially at high ambient temperatures (Zhuge and Klopfenstein, 1986), high temperature exposure during pelleting (Yang et al., 2020), and exposure to inorganic trace minerals (Shurson et al., 2011) in premixes or complete feeds. Finally, vitamin excesses are generally well-tolerated by poultry, and even with several-fold excesses, the risk of hypervitaminosis is low when supplemented at commercially relevant levels of inclusion (National Research Council, 1987). Based on what is known at the present time, the over-supplementation of vitamins comes at a small cost, with very low risks to health and productivity.

## INTERACTIONS BETWEEN VITAMINS AND THE GUT MICROBIOTA

The gastrointestinal tract is one of the largest organ systems of the body, and has high metabolic and nutrient demands. It is also home to the largest microbial ecosystem in the body. The intestinal microbiota is an important utilizer of consumed nutrients, and can also contribute nutritionally relevant amounts of nutrients and biofactors that can contribute to the nutrition and health of the host. With a few exceptions, the interaction between the poultry intestinal microbiota and vitamin nutrition of the host is not well-studied in poultry. Therefore, many of the concepts discussed in this review are based on the mammalian literature. Specific examples from poultry nutrition will be included where possible.

Low intakes of various vitamins can alter the human gut microbiome, with subsequent detrimental effects on host health. Although there is little data on the direct utilization of vitamin D in the metabolism of intestinal bacteria (Yamamoto and Jorgensen, 2019), vitamin D intake in humans is negatively associated with the abundance of *Prevotella*, and positively associated with abundance of *Bacteroides* (Wu et al., 2011). Eight weeks of vitamin D supplementation increased human upper gastrointestinal tract microbiome richness, along with decreased presence of Proteobacteria and increased Bacteroidetes; microbial composition of the lower gastrointestinal tract was not affected (Bashir et al., 2016). Vitamin D also has direct effects on intestinal health by maintaining tight junction integrity, direct and indirect modulation of inflammation and production of antimicrobial peptides (Akimbekov et al., 2020). Additionally, exposure to narrow-band ultraviolet light increased serum 25-hydroxy vitamin D levels, and increased fecal microbiota diversity of humans not taking an oral vitamin D supplement, but not in those taking a supplement (Bosman et al., 2019). The authors suggested this may have a positive effect on the severity of diseases associated with low vitamin D status such as inflammatory bowel disease and multiple sclerosis. Compared to deficiencies of folate, iron and zinc, an acute vitamin A deficiency in gnotobiotic mice gavaged with human gut microbiota (defined 92 strain culture) had the largest impact on the microbial community and metatranscriptome (Hibberd et al., 2017). DL-α-tocopherol supplementation to mice decreased the ratio of Firmicutes to Bacteroides in the cecal microbiota (Choi et al., 2020). Supplementation of vitamin E as tocotrienol altered the gut microbiome and increased glucose metabolism and reduced markers of inflammation in mice fed a high-fat diet (Chung et al., 2020). Supplementation of synthetic vitamin B_12_ (cyanocobalamin) increased the presence abundance of *Enterobacteriaceae* and aggravated symptoms of inflammatory bowel disease in mice (Zhu et al., 2019), but methylcobalamin (naturally occurring form) increased production of butyrate and propionate in vitro (Xu et al., 2018), which may alleviate intestinal inflammation in vivo. Although these studies show that the relationship between the microbiota and the host involves vitamins, it is not yet possible to strategically use this knowledge for the advantage of poultry health and productivity.

### Theoretical Contribution of the Microbiome to Intestinal Nutrient Supply

Magnúsdóttir et al. (2015) estimated the theoretical production of eight B-vitamins by the human gut microbiota, based on genomic evaluation of the biosynthetic pathways for each present in the microbiome. Based on that data, they determined the proportion of microbial species capable of producing each of the vitamins, and the amount that could be released upon microbial cell death within the digestive tract. They then calculated the proportion of the human daily recommended intake for each of the vitamins that could be supplied via intestinal microbial biosynthesis. A similar study has not been reported in poultry; however, if the requirements for each of the vitamins are assumed to be somewhat similar between humans and poultry, and assuming a similar capacity for vitamin synthesis by the poultry microbiome, comparisons of the relative importance of microbial synthesis vs. typical commercial dietary vitamin supply can be made. Table 2 shows the theoretical production of B vitamins by the human intestinal microbiome from Magnúsdóttir et al. (2015). In that context, the potential contribution of the poultry microbiome was estimated for laying hens, broilers, and turkeys. This approach necessarily required several important assumptions.

1. The capacity of the poultry gut microbiome to produce vitamins is relatively consistent with that of the human intestinal microbiome.
2. The expression of microbial vitamin synthesis as a proportion of the daily recommended intake (**DRI**) for humans can be extrapolated to the proportion of the vitamin requirements of poultry expressed as a percentage of the requirements on a mg/kg feed basis.
3. The inclusion of requirements for mature laying hens, for broilers over 25 days of age and turkeys over 18 wk of age reflect the oldest age categories provided in the commercial recommendations (DSM Nutritional Products, 2016), and therefore a mature laying hen microflora (Xiao et al., 2021) and a relatively stable, if not completely mature, broiler (Lu et al., 2003; Jurburg et al., 2019) and turkey (Morishita et al., 1992) gut microflora.
4. The NRC (1994) requirement values are based on total dietary inclusion; the commercial recommendations (DSM Nutritional Products, 2016) are for dietary supplementation, and do not account for vitamin content of the feeds before vitamin supplementation.
5. Inefficiencies in the extent of absorption of microbially-synthesized vitamins were not accounted for in these theoretical calculations.

**Table 2:** Theoretical B-vitamin production by the human intestinal microbiome (Magnúsdóttir et al., 2015), and corresponding estimates of microbial and dietary vitamin supply in poultry.

Vitamin	Human microbiome	NRC requirement,mg/kg feed^2^			Estimated microbial production, equivalent to mg/kg feed^3^			Commercial requirement, mg/kg feed^2^			Estimated poultry microbiome production; % of commercial dietary recommendation^4^		
	% of DRI^1^	Laying hen	Broiler chicken	Turkey	Laying hen	Broiler chicken	Turkey	Laying hen	Broiler chicken	Turkey	Laying hen	Broiler chicken	Turkey
Thiamine	2.3	0.6	1.8	2	0.014	0.041	0.05	2.5–3.0	2–3	2–3	0.460	1.380	1.53
Riboflavin	2.8	2.1	3.6	2.5	0.059	0.101	0.07	5–7	6–8	8–10	0.840	1.260	0.70
Niacin	27	8.3	30	40	2.241	8.100	10.80	30–50	50–80	50–60	4.482	10.13	18.0
Pantothenate	0.078	1.7	10	9	0.001	0.008	0.01	8–12	10–15	15–20	0.011	0.052	0.04
Pyridoxine	86	2.1	3.5	3	1.806	3.010	2.58	3.5–5	4–6	3–6	36.12	50.17	43.0
Biotin	4.5	0.08	0.15	0.1	0.004	0.007	0.01	0.10–0.150	0.25–0.40	0.20–0.25	2.400	1.688	1.80
Folacin	37	0.21	0.55	0.7	0.078	0.204	0.26	1.0–1.5	2–2.5	2–2.5	5.180	8.140	10.36
Vitamin B_12_	31	0.004	0.01	0.003	0.001	0.003	0.001	0.15–0.25	0.020–0.030	0.15–0.25	4.960	10.333	3.72

1 Theoretical % of daily recommended intake (human) produced by microbiome (Magnúsdóttir et al., 2015).

2 Laying hen consuming 100 g/day; broiler finisher diet (25+ days of age) and turkey finisher diet (18+ wk of age).

3 Calculated assuming a similar proportion of the total daily vitamin requirement is produced by the poultry microbiome as by the human microbiome.

4 Calculated based on the commercial recommendations provided in Table 1. When a range is given in the table; the higher value was used to calculate the %microbial production relative to dietary supplementation.

It is difficult to prove that these assumptions are completely accurate, and the table is not intended as a definitive quantification of microbial vitamin synthesis in poultry. Rather, it is intended to put potential microbial vitamin production (relatively minor in most cases) in the context of commercial levels of vitamin supplementation (generally provided far in excess of the minimum requirement). The calculated values for microbial synthesis of pyridoxine range from 26 to 50% of recommended dietary supplementation levels for layers, broilers and turkeys. There are substantial differences in the NRC recommendations for niacin among layers, broilers and turkeys (8.3, 30, and 40 mg/kg diet, respectively), but the commercial recommendations are more similar (50, 80, and 60 mg/kg diet, respectively). Even with a relatively constant proportion of microbial niacin synthesis, the turkey microbiome would synthesize an amount of niacin equivalent to 18% of the recommended dietary supplementation. In the case of the remaining vitamins, the calculations in Table 2 suggest that microbial vitamin synthesis would account for at most, approximately 10% of the recommended supplemental level, and in several cases, less than 1%. B vitamins synthesized by the mammalian microbiome are largely utilized within the gut by other (non-vitamin producing) microbes (Yoshii et al., 2019; Lauridsen et al., 2021). Therefore, the impact of competition for microbially-synthesized vitamins on the quantity of vitamins that can be absorbed by the host is unknown. Because of retrograde peristalsis in the avian digestive tract (Brummermann and Braun, 1995), intestinal absorption of microbially synthesized vitamins from the ceca may be more efficient in poultry than in pigs or humans.

Although vitamin K synthesis takes place in the intestine of the chick, it is not sufficient to meet the requirement (Nelson and Norris, 1961b). Addition of the antibiotic sulfaquinoxaline to the diet of chicks increased the vitamin K requirement by 4- to 7-fold relative to unmedicated chicks (Nelson and Norris, 1961a). In pheasant chicks, sulfaquinoxaline increased the vitamin K requirement by approximately 8-fold. Therefore, although microbial synthesis alone is not sufficient to meet the complete vitamin K requirement, alteration of the gut microflora by antibiotics can substantially reduce vitamin K synthesis in poultry.

Vitamin synthesis pathways enriched by a dietary *Macleya cordata* extract included those for pyridoxine, riboflavin, vitamin K and vitamin E, demonstrating that the capacity of the chicken gut microbiome to synthesize vitamins can be modulated by the diet (Huang et al., 2018). Maximum metabolic capacity of the microbiome was reached between 15 and 28 days of age (Huang et al., 2018).

It is unknown how over-supplementation of dietary vitamins influences the gut microbiome, and whether commercial levels of dietary vitamins are optimal for microbiota and intestinal health in poultry. From a performance standpoint, commercially relevant levels of vitamins are well-tolerated by poultry (National Research Council, 1987). It remains to be seen whether these levels have a beneficial, neutral, or detrimental effect on the intestinal microbiome.

## BIOFACTOR INTERACTIONS BETWEEN THE HOST AND MICROBIOTA

### Biofactors

Biofactors are compounds that can regulate or influence biological functions. This definition includes molecules not synthesized by the body such as vitamins, but also microbial metabolites that influence gut metabolism and physiology. The synthesis of biofactors by the intestinal microbiome is extensive (Albenberg and Wu, 2014; De Angelis et al., 2019), and it is beyond the scope of this review to give an exhaustive overview of biofactors in poultry diets. Two examples of biofactors arising from the microbial action in the gut of poultry are myo-inositol from the degradation of phytate, and butyrate (among other products) arising from the fermentation of soluble fibers in the ceca.

#### Myo-inositol

Myo-inositol is synthesized from glucose within the body in mammals (Burton and Wells, 1974; Clements and Diethelm, 1979), as well as the chicken (Kanehisa et al., 2017; Kanehisa et al., 2019; Gonzalez-Uarquin et al., 2020). It is also the end product of complete degradation of dietary phytic acid (myo-inositol 1,2,3,4,5,6-hexakis (dihydrogen phosphate) by phytases or phosphatases in the intestine. The enzymes responsible for phytate degradation can be intrinsic (contained in the plant-based feed ingredients) (Leytem et al., 2008), endogenous (host-derived) (Sommerfeld et al., 2019), intestinal microbiome-derived (Borda-Molina et al., 2016), or exogenous (as microbially-derived commercial phytase products) (Bello et al., 2019; Pongmanee et al., 2020; Al-Qahtani et al., 2021). As the digestive tract matures, the capacity for dietary phytate degradation increases, even in the absence of exogenous phytase. Myo-inositol is absorbed from the diet by birds (Lee and Bedford, 2016; Sommerfeld et al., 2018), but the relative contribution of myo-inositol absorbed from the digestive tract on the amount of myo-inositol available for use in the body is unknown (Gonzalez-Uarquin et al., 2020).

Myo-inositol influences many physiological systems, increasing mineral absorption, bone mineralization, skeletal muscle glucose uptake and breast muscle development in broilers, male reproduction, influencing neuronal signaling, and decreasing feather pecking and aggressive behavior in laying hens (reviewed by Gonzalez-Uarquin et al., 2020). Within the chicken, myo-inositol may act as a growth promotor (Hegsted et al., 1941; Cowieson et al., 2013; Cowieson and Zhai, 2021). As myo-inositol's role in metabolic regulation and productivity becomes more clearly understood, the ability to strategically supplement or target myo-inositol metabolism may represent an opportunity to more effectively manage bird health and productivity.

Intestinal bacteria capable of metabolizing myo-inositol include *Clostridium* (Kawsar et al., 2004), *Salmonella* (Hellinckx et al., 2017), and *Lactobacillus* (Yebra et al., 2007; Zhang et al., 2010) species. Myo-inositol may also serve as a precursor for microbial short-chain fatty acid synthesis within the intestinal tract. Dietary supplementation of sodium phytate and myo-inositol increased cecal butyrate production in the rat, and decreased serum proinflammatory cytokine levels (Okazaki and Katayama, 2014). As with the vitamins, the interplay between microbial production and use of myo-inositol in the context of supply to the host is not well understood, nor is the impact of increasing the availability of myo-inositol within the intestine of poultry on gut health.

#### Butyrate From the Fermentation of Soluble Fiber

Historically, poultry nutritionists have mainly viewed dietary fiber as an antinutritional factor. Structural, insoluble carbohydrate (e.g., cellulose and some hemicelluloses) are largely unaffected by microbial degradation in the poultry gut because of the short residence time and small volume of the ceca. Apparent total tract fiber digestibility of diets having a range of insoluble non–starch polysaccharides (wheat bran and corn) was low, ranging from 8 to 19% (Jørgensen et al., 1996; Meng and Slominski, 2005). Increasing dietary crude fiber content decreased nutrient density, resulting in increased feed intake and feed conversion ratio (Röhe et al., 2019; Nascimento et al., 2020; Sozcu and Ipek, 2020; Tejeda and Kim, 2020). Soluble fibres (e.g., arabinoxylans, β-glucans, and some hemicelluloses) increase digesta viscosity and interfere with nutrient digestion and absorption and energy yield from feedstuffs (Choct and Annison, 1990). The apparent total tract fiber digestibility of diets high in soluble non–starch polysaccharides (barley, wheat and oats) was higher than for insoluble fibre, ranging from 28 to 40% (Pettersson and Aman, 1989; Jørgensen et al., 1996).

The impact of microbial fiber degradation has received more attention in pigs than in chickens. Fiber fermentation can provide 17% of the daily energy requirement of growing pigs, and 25% of the energy requirement of sows (Iyayi and Adeola, 2015), but only 3 to 4% in broiler chickens (Jørgensen et al., 1996). However, more recently, the role of fermentable carbohydrates in poultry have begun to receive more attention because of their potential beneficial effect on intestinal health, resistance to pathogens, and poultry productivity.

The general effects of hindgut fermentation of carbohydrates in monogastrics have been recently reviewed (Adebowale et al., 2019; Tiwari et al., 2019 Bendiks et al., 2020). Outcomes include increased tight junction integrity in the gut, thereby reducing gut leakage and translocation of intestinal bacteria, enrichment of specific types of bacteria purported to have beneficial effects on host health, and increased short-chain fatty acid production. Short-chain fatty acids, particularly butyrate increase gut integrity, act as a fuel for intestinal cells, reduce gut inflammation, and participate in systemic regulation of immune function and appetite, and reduce hindgut pH, which reduces populations of pathogenic bacteria (Bach Knudsen et al., 2018).

Prebiotics are indigestible dietary ingredients that can be broken down by specific groups of bacteria considered to be beneficial to bird health, allowing them to outcompete pathogens that are not able to utilize the prebiotic (Yaqoob et al., 2021). The prebiotics may be preferentially used by butyrate-producing bacteria (Scott et al., 2015; Patrascu et al., 2017). Various carbohydrates, primarily oligosaccharides are used in poultry diets as prebiotics. Mannanoligosaccharides (Pourabedin et al., 2017; Praxedes-Campagnoni et al., 2021), xylo-oligosaccharides (Pourabedin et al., 2017), fructo-oligosaccharides (Xu et al., 2003), galacto-oligosaccharides (Hughes et al., 2017), and yeast β-glucans have each shown promise to modulate the intestinal microbiota and reduce pathogen colonization.

## KNOWLEDGE GAPS

### Vitamins, Gut Health, and Productivity

The relatively low cost of supplementation and the high tolerance for excess dietary vitamins has led to a dearth of vitamin nutrition requirement studies in high-producing modern laying hens, broiler chickens, and turkeys. Changes in absorptive efficiency and metabolism may have been an inadvertent consequence of selection for feed efficiency, but this has not been studied. Therefore, although we know that commercial poultry diets contain vitamins well above the NRC (1994) requirements, it is not known how great an excess is supplied relative to the actual requirements of modern genotypes fed practical diets. In this context, it is difficult to assess the economy of intestinal microbiome production and consumption of vitamins. Given these knowledge gaps, it is difficult to predict whether strategic changes in our approach to vitamin supplementation, including targeted delivery of vitamins to the site of absorption or protection from use by the gut microflora will influence bird health and productivity, or the health of the intestinal microbiome itself.

### Biofactors

Given some of the positive effects observed with dietary myo-inositol supplementation, understanding how to liberate additional myo-inositol from the diet, its effect on the microbiome and its absorption and utilization by the bird is a logical extension of phytase research. Questions still remain regarding the optimum means of providing short-chain fatty acids, and butyrate specifically to the intestinal tract environment. A greater understanding of the microbiome will allow nutritionists to formulate diets that will favor butyrate producing microbial species, and provide appropriate substrate to optimize butyrate production.

With increasing research into the interaction between the metabolome and the host, it is likely that the ability to fine-tune dietary composition to optimize microbiome function to the benefit of host health, intestinal function, and poultry productivity.

## DISCLOSURES

The author does not have any conflicts of interest to declare.
